# Testing Needed for Acesulfame Potassium, an Artificial Sweetener

**DOI:** 10.1289/ehp.114-a516a

**Published:** 2006-09

**Authors:** Myra L. Karstadt

**Affiliations:** Department of Environmental and Occupational Health, Drexel University School of Public Health, Philadelphia, Pennsylvania, E-mail: myrakarstadt@verizon.net

In their article “First Experimental Demonstration of the Multipotential Carcinogenic Effects of Aspartame Administered in the Feed of Sprague-Dawley Rats,” Soffritti et al. (2006) present interesting data on the carcinogenic effects of long-term exposure to aspartame, an artificial sweetener, in experimental animals (rats).

Recently, aspartame was supplanted as the leading artificial sweetener by sucralose, marketed in the United States under the trade name Splenda (McNeil Nutritionals, LLC, Ft. Washington, PA). As of 2005, Splenda was reported to have > 50% of the market for artificial sweeteners, while aspartame [Equal (Merisant Company, Chicago, IL); NutraSweet (NutraSweet Property Holdings Inc., Chicago, IL)] had < 20% (Associated Press 2005). Splenda is typically used in sweetener blends, most frequently with acesulfame potassium (CAS RN 55589-62-3) (Sunett; marketed in the United States by Nutrinova, Somerset, NJ).

The Food and Drug Administration’s (FDA) multiple approvals of food additive petitions (FAPs) for acesulfame began in 1988 (FDA 1988), and culminated in 1998 with approval of the use of acesulfame in soft drinks (FDA 1998), historically the largest single use of artificial sweeteners. All of the FDA’s approvals of FAPs for acesulfame were grounded on the conclusion that safety studies, including long-term animal tests of acesulfame carried out for Hoechst, the manufacturer of the chemical, in the Netherlands in the 1970s, were adequate and the test results indicated safety.

The 1970s tests of acesulfame—two tests carried out in rats and one in mice—are inadequate to establish lack of potential carcinogenicity. Here are a few reasons why the tests are inadequate [Center for Science in the Public Interest (CSPI) 1996]:

- Subchronic tests were not conducted for the rats and mice used in the tests on which the FAPs rested
- It is likely the minimum toxic dose/maximum tolerated dose (MTD) was not achieved in the rat and mouse studies
- Randomization of test groups was not carried out properly
- Mice were held on test for only 80 weeks, rather than the 104 weeks characteristic of National Toxicology Program (NTP) bioassays
- Animal husbandry and monitoring of animals on test were evidently poor, as indicated by high disease rates in the animals and extensive autolysis of tissues.

Even with the flaws in design and execution of the Hoechst tests, results indicated an association between treatment with acesulfame and carcinogenesis (CSPI 1996).

Working-level staff members at the FDA identified deficiencies in the acesulfame tests in the 1980s (McLaughlin 1986; Taylor 1986). Thus, an FDA staff member (Taylor 1986) noted in 1986, when the FDA had decided to accept the Hoechst studies, that

The question remains whether these studies are sufficiently definitive or rigorous in light of the potential for widespread, [sic] high exposure to acesulfame K in all group [sic] in the population.

In 1996, the CSPI nominated acesulfame for testing in the NTP bioassay program (CSPI 1996), and provided the NTP with detailed information on the Hoechst tests and their flaws. Although an individual familiar with test design and implementation could have concluded with ease that the Hoechst tests were not consistent with the criteria established for NTP bioassays or the test guidelines set out in the FDA’s *Redbook* (FDA 1982), and that it was likely that, at some point, many people would be exposed to acesulfame, the NTP rejected CSPI’s nomination.

In 2003, the NTP announced the results of tests of both aspartame and acesulfame in genetically modified mice (GMM) (NTP 2005). Both chemicals gave negative results in the tests, carried out in two strains of GMM.

The NTP’s final report on those GMM studies (NTP 2005) noted that aspartame and acesulfame had been selected as “negative controls” for validation tests for the GMM models. The chemicals did indeed test negative, but that negative result did not advance our understanding of potential carcinogenicity of acesulfame. Regarding the GMM tests of aspartame and acesulfame, Martha Sandy of the California Environmental Protection Agency, stated that

[N]egative results [in the GMM tests] are not informative as to the test substance’s carcinogenicity, and point to the need to conduct standard two-year carcinogenicity studies. At this time, transgenic models cannot replace the two-year bioassay and it would be unwise to list a chemical as safe for human exposure based upon negative results in not yet validated model systems. (Sandy 2003)

The findings of Soffritti et al. (2006) of multipotential carcinogenesis in rats fed aspartame over their lifetimes provide support for Sandy’s (2003) statements.

I have sent the NTP a new nomination of acesulfame for 2-year bioassay testing (Karstadt 2006).
